# Investigating association of triglycerides with hemodynamic parameters in patients with Low cardiovascular risk using 4D flow MRI

**DOI:** 10.3389/fcvm.2026.1826181

**Published:** 2026-06-24

**Authors:** Weiling Wang, Shuangqi Fu, Yong Xiang, Yingjiao Shen, Wei Wang, Jianyu Li, Ling Luo

**Affiliations:** Department of Radiology, Guiqian International General Hospital, Guiyang, Guizhou, China

**Keywords:** carotid artery (CA), four-dimensional magnetic resonance flow imaging (4D flow MRI), MRI, triglycerides (TG), wall shear stress (WSS)

## Abstract

**Introduction:**

Cardiovascular disease (CVD) remains a global health challenge, with hypertriglyceridemia as a key risk factor. The relationship between triglycerides (TG) and carotid hemodynamics in low-risk populations is unclear.

**Materials and methods:**

This prospective study enrolled 180 participants aged 55 years or younger with low cardiovascular risk. Participants were divided into three groups based on TG levels: normal TG (<1.7 mmol/L), elevated TG (1.7–5.64 mmol/L), and severely elevated TG (≥5.65 mmol/L). 4D Flow MRI data were acquired using a 3 T MRI scanner, and hemodynamic parameters were measured. Statistical analysis included the Mann–Whitney *U*-test, Kruskal–Wallis *H*-test, ANOVA, mixed-effects models, and multiple linear regression.

**Results:**

A total of 106 participants (mean age 37.05 ± 8.17 years, 62 males) were included. Significant differences in WSS-related parameters, particularly WSSmax and WSSmean, were observed across TG groups. In the RICA-1 segment, for normal TG, WSSmax (0.29 ± 0.10 Pa) and WSSmean (0.20 ± 0.06 Pa) were similar to those in the elevated TG group (WSSmax: 0.29 ± 0.10 Pa, WSSmean: 0.20 ± 0.06 Pa). In the severely elevated TG group, WSSmax (0.44 ± 0.14 Pa) and WSSmean (0.28 ± 0.08 Pa) were significantly higher than in the other groups (*p* < 0.001). Similar differences were observed in the LICA-1 segment. In mixed-effects models adjusting for age, sex, total cholesterol, HDL-C, BMI, and smoking status, TG remained independently associated with WSSmax (B = 0.010, *p* < 0.001) and WSSmean (B = 0.004, *p* = 0.004).

**Conclusion:**

Our study demonstrates that TG levels are independently associated with carotid WSS parameters after comprehensive covariate adjustment, with the most pronounced changes in the ICA-1 region among individuals with severely elevated TG.

**Clinical Trial Registration:**

https://www.chictr.org.cn/showprojEN.html?proj=224462, ChiCTR2400082465.

## Introduction

Cardiovascular disease (CVD) remains a significant global health challenge, with its development intricately linked to a multitude of risk factors (1). Accumulating evidence highlights that cumulative lifetime exposure to lipids significantly increases the risk of cardiovascular (CV) events (2). Notably, nearly half of patients with ischemic stroke are affected by dyslipidemia (3, 4). A comprehensive review by Simha highlighted the multifactorial etiology of hypertriglyceridemia, including genetic lipid metabolism disorders, glycogen storage diseases, and lifestyle factors (5). A recent European Atherosclerosis Society consensus statement by Ginsberg et al. further established that triglyceride-rich lipoproteins (TRL) and their remnants can penetrate the arterial wall, induce endothelial dysfunction, and promote local inflammation, thereby contributing directly to vascular pathophysiological changes (6). In addition, Liang et al. specifically demonstrated that hypertriglyceridemia may increase the risk of ischemic stroke not only through atherosclerosis and thrombosis, but also by increasing blood viscosity and thereby affecting hemodynamics (7). As a vital component of the systemic arterial system, the carotid artery is highly sensitive to early vascular functional alterations and serves as an indicator of hemodynamic changes.

Recent advances in imaging technology have led to the development of four-dimensional magnetic resonance flow imaging (4D Flow MRI), which allows for comprehensive characterization of cardiovascular blood flow in a single acquisition. This technique surpasses traditional 2D flow imaging in terms of data evaluation flexibility, valve tracking, and comprehensive flow assessment (8). In intracranial vascular disease, 4D Flow MRI has been employed to assess hemodynamic changes in conditions such as intracranial aneurysms and atherosclerosis, providing valuable insights into disease progression and risk assessment. Similarly, in carotid artery disease, 4D Flow MRI has been used to investigate the relationship between carotid atherosclerotic plaques and hemodynamics (9, 10).

Current guidelines for dyslipidemia management, established by various countries, primarily focus on cardiovascular disease risk (11–13), with recommendations largely derived from long-term cohort studies conducted in diverse populations (14). However, these guidelines offer limited advice on managing hypertriglyceridemia in low cardiovascular risk populations, particularly regarding carotid artery health. Given the high prevalence of dyslipidemia among Chinese adults—reported at 35.6% in individuals aged ≥18 years in a 2018 national survey (15)—there is an urgent need for robust evidence to guide the clinical management of hypertriglyceridemia in this population.

4D Flow MRI offers the potential to detect subtle changes in early carotid hemodynamics among hypertriglyceridemic patients by measuring multidimensional flow parameters. Investigating these hemodynamic changes could provide novel insights into the relationship between hypertriglyceridemia and carotid artery function. Therefore, the aim of this study is to preliminarily assess carotid hemodynamic changes in hypertriglyceridemic patients with low cardiovascular risk using 4D Flow MRI.

## Materials and methods

### Participants

**Figure 1:**
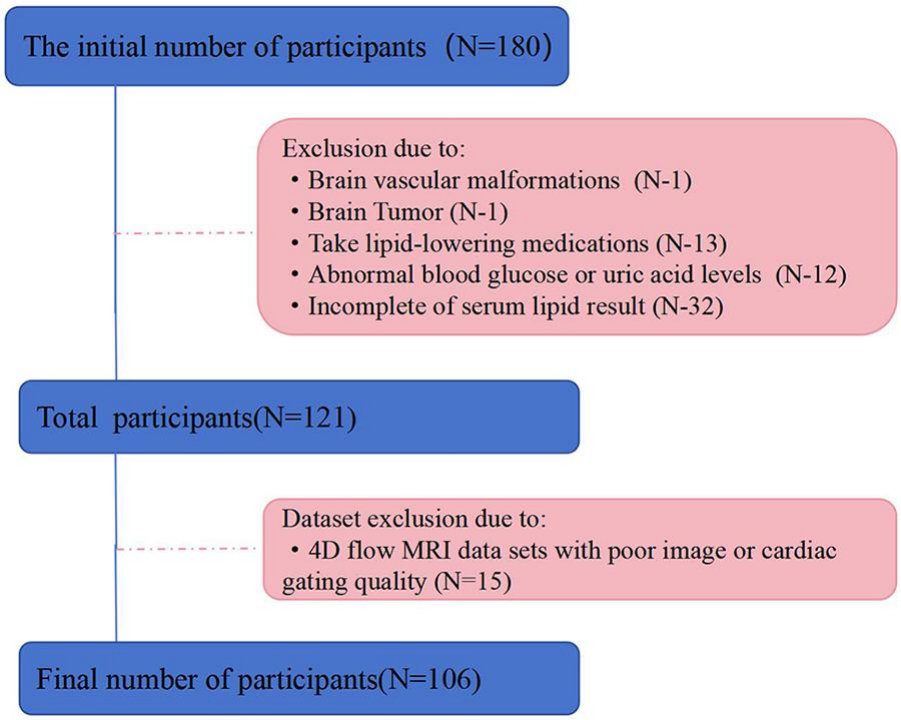
Flowchart of participant inclusions and exclusions.

This observational prospective study adhered to the 2013 Declaration of Helsinki and was approved by the Ethics Committee of Guiqian International General Hospital. All participants were informed of the study objectives, procedures, and potential risks, and written informed consent was obtained from each participant prior to enrollment. The study was also registered with the Chinese Clinical Trial Registry under registration number ChiCTR2400082465. Between April 2024 and November 2024, we recruited 180 participants from the health examination center of our hospital. Since aging has been shown to influence hemodynamic parameters in middle-aged and elderly individuals (16), participants were restricted to those aged 55 years or younger. Venous blood samples were collected after an 8-hour fast to measure serum triglycerides (TG), total cholesterol (TC), and high-density lipoprotein cholesterol (HDL-C). Participants were assessed using the China-PAR cardiovascular risk assessment tool (17), and only those with a low 10-year cardiovascular risk (<10%) were included in the study. During the examination, Some patients were excluded based on medical history, including those with clinically diagnosed diabetes or gout, as both conditions are known to independently affect endothelial function and hemodynamics (18, 19), as well as individuals with cerebrovascular malformations, brain tumors, lcontinuous use of lipid-lowering medications for ≥3 months before enrollment, incomplete serum lipid results, or poor image quality on 4D flow MRI. Of the 180 participants enrolled, 32 were excluded due to incomplete lipid panels resulting from initial coordination gaps with the health examination center, which were identified and corrected midway through the study. An additional 15 participants were excluded because their 4D Flow MRI datasets became incompatible with the post-processing software (CVi42) following an unanticipated scanner system upgrade; these exclusions were purely technical. Furthermore, as the post-processing software was located at an external institution requiring on-site travel, budgetary constraints prevented additional processing sessions. No participants were selectively removed based on data characteristics. This sample attrition is acknowledged as a limitation. The recruitment process is shown in Figure 1.

Based on fasting TG levels, participants were further stratified into the following groups:

### Normal TG group (<1.7 mmol/L)

Defined per the Lancet large-scale study (Sarwar N et al., 2010), this range correlates with stable lipid metabolism and minimal cardiovascular risk (11).

### Elevated TG group (1.7−5.64 mmol/L)

Aligned with American Heart Association criteria, this interval indicates lipid metabolism imbalance and elevated cardiovascular risk (13).

### Severely elevated TG group (≥5.65 mmol/L)

Adopted from the Chinese Lipid Management Guidelines' pancreatitis intervention threshold, this level confers significant pancreatitis and metabolic disorder risks (15).

### MRI acquisition

A United Imaging 3T 880 MRI scanner, paired with a 48-channel head and neck coil, was used to acquire four—dimensional flow MRI data. The 4D Flow sequence employed a non-contrast-enhanced, radially undersampled phase-contrast technique combined with isotropic projection reconstruction. The detailed protocol parameters were as follows: the repetition time (TR) was 20.7 ms, the echo time (TE) was 2.77 ms, the flip angle was 10°, the spatial resolution was 1.39 × 1.39 × 2.0 mm, the readout resolution was 192, the phase resolution was 100, and the velocity encoding (VENC) values in the slice, phase, and readout directions were all set at 100 cm/s. The scan duration was approximately 15 min. Retrospective pulse oximetry gating was used to reconstruct the velocity, magnitude, and phase data into 20 cardiac time frames that were evenly distributed throughout the cardiac cycle for each patient.

### Image processing

**Figure 2:**
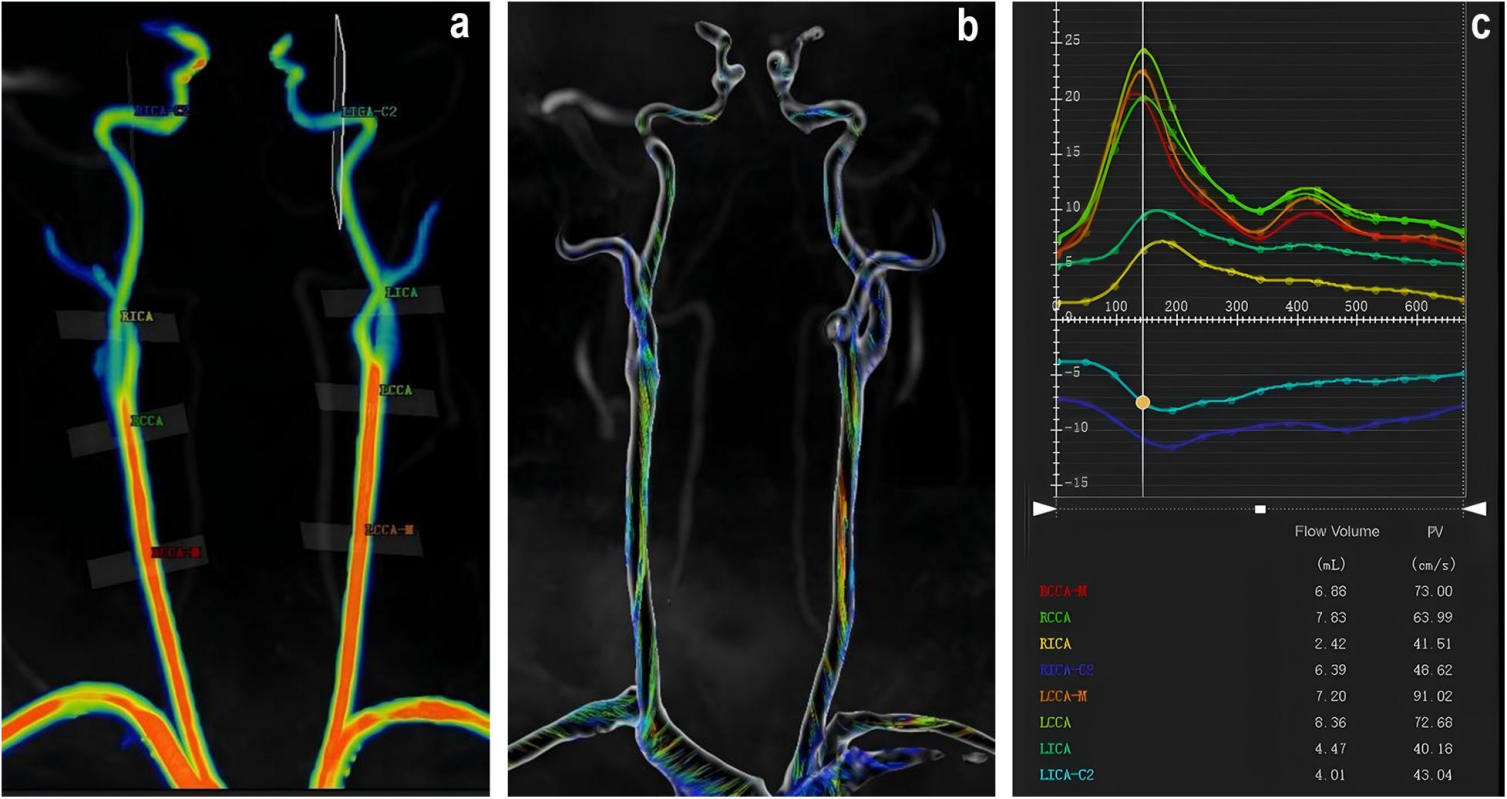
Hemodynamic assessment of carotid arteries using 4D flow MRI. **(a)** Schematic representation of the eight manually selected vascular segments for analysis: the mid-segment (CCA-M) and distal segment (CCA) of the common carotid artery, and the proximal (ICA-1) and distal (ICA-2) segments of the internal carotid artery, for both the left and right sides. These regions were visualized in three dimensions to enable precise hemodynamic measurements. **(b)** Representative 3D streamlines depicting blood flow patterns within the carotid arteries. **(c)** Quantitative hemodynamic parameters measured in each vascular segment, including flow volume and peak velocity (PV).

Post-processing was performed using CVi42 4D Flow v5.14 software, involving semi-automated steps: global thresholding, centerline skeletonization, orthogonal plane generation, and in-plane k-means clustering segmentation. Four anatomical segments per side—mid and distal common carotid artery (CCA-M, CCA), proximal and distal internal carotid artery (ICA-1, ICA-2)—were manually selected from the 3D interface (Figure 2), yielding eight segments per subject. Hemodynamic parameters (flow volume, peak velocity, average axial/circumferential WSS, WSSmean, WSSmax) were extracted for each segment. To capture systemic hemodynamic trends, segment-level parameters (e.g., WSSmax) were arithmetically averaged across all eight segments. This averaging approach mitigated spatial heterogeneity and enhanced statistical power for population-level analyses. Two observers independently performed the analysis: Observer 1 (W, with 6 years of experience in radiology) and Observer 2 (F, with 10 years of experience in radiology). To test inter-observer reproducibility, Observer 2 repeated the measurements on the same 30 randomly selected datasets one month after the initial analysis, without access to the initial results. Bland-Altman plots were performed to visually check agreement, and the intraclass correlation coefficient (ICC) was calculated using a two-way random effects model for absolute agreement.

### Statistical analysis

Statistical analyses were performed using SPSS (version 26.0; IBM Corp), and figures were generated with R software (version 4.2.0). Baseline characteristics of study participants were expressed as counts (percentages) for categorical variables. Continuous variables with normal distribution were reported as mean ± standard deviation (SD), while those with non-normal distribution were reported as median (interquartile range). Normality of continuous variables was assessed using the Shapiro–Wilk test. For comparisons among the three groups, one-way analysis of variance (ANOVA) was used for normally distributed data, followed by *post-hoc* tests with Bonferroni correction. The Kruskal–Wallis *H*-test was used for non-normally distributed data, followed by Dunn's *post-hoc* test for multiple comparisons. Comparisons between any two specific groups were performed using the independent samples *t*-test for normally distributed data or the Mann–Whitney *U*-test for non-normally distributed data.

To evaluate the independent association between triglycerides and hemodynamic parameters while accounting for potential confounders, we implemented two complementary multivariable approaches. First, multiple linear regression models were fitted using per-participant averaged values of hemodynamic parameters across the eight vascular segments. These models included sex, age, TG, TC, HDL-C, BMI, and smoking status as fixed covariates. Second, linear mixed-effects models were fitted on segment-level data (eight segments per participant) to retain within-participant spatial information. The models included the same fixed effects as the regression models, a random intercept for participant ID to account for within-participant correlation, and vascular segment as a repeated measure with a diagonal covariance structure. Model parameters were estimated using restricted maximum likelihood (REML) with Satterthwaite approximation for denominator degrees of freedom. Statistical significance was defined as *p* < 0.05. All reported *p*-values are two-sided.

For exploratory group comparisons across the eight vascular segments and six hemodynamic parameters (48 tests), the Benjamini-Hochberg false discovery rate (FDR) procedure was applied to control for multiple testing. Sensitivity analysis excluding BMI and smoking showed minimal changes in TG coefficients (e.g., WSSmax B changed from 0.010 to 0.011), supporting robustness. Propensity matching was not feasible given the small sample size.

## Results

### Participants' characteristics and intergroup differences

**Figure 3:**
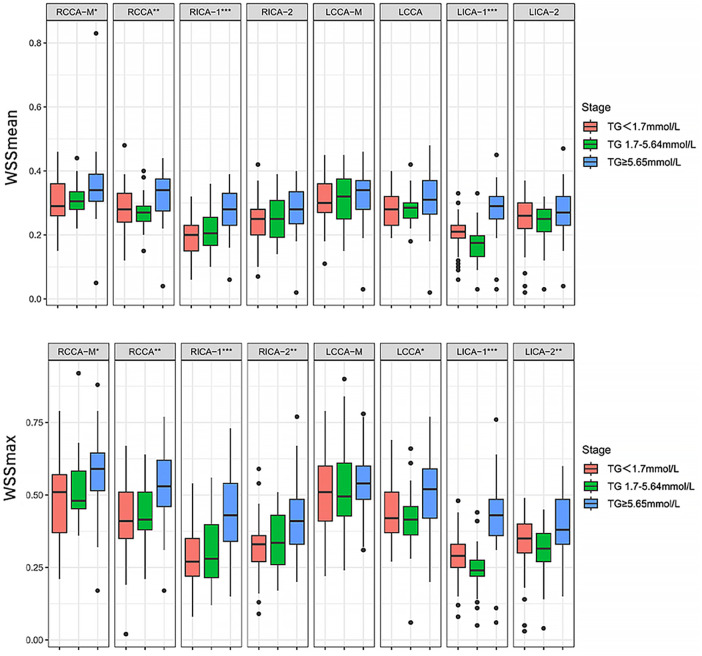
Statistical comparison of WSSmean and WSSmax between groups for different carotid artery segments. The number of asterisks in the figure indicates the statistical significance of differences in WSSmean and WSSmax values between the three groups across carotid segments (*P ≤ 0.05, **P < 0.01, ***P < 0.001). The most pronounced differences (***) were observed in bilateral internal carotid artery (ICA-1) segments, particularly in the group with severely elevated TG levels.

A total of 106 participants were included in the final analysis: 59 in the normal TG group, 20 in the elevated TG group, and 27 in the severely elevated TG group. Participant demographics and baseline characteristics are shown in Table 1. Significant differences were observed between the three groups in terms of age, gender, body mass index (BMI), and smoking history (all *p* < 0.001), as detailed in Table 1. Post-processing of imaging for each participant took approximately 5 min, and eight vascular segments were selected for measurement per participant, resulting in a total of 848 vascular segments from which 4D Flow MRI hemodynamic parameters were extracted (detailed data are provided in Supplementary Table S1). After FDR correction for multiple comparisons, significant intergroup differences remained in WSSmax and WSSmean at the bilateral ICA-1 segments, as well as in WSSmax at multiple other segments. Significant intergroup differences were observed in WSSmean and WSSmax at the initial segment of the bilateral internal carotid arteries (ICA-1), particularly in the severely elevated TG group (FDR < 0.05 for all; see Figure 3 and Supplementary Table S1). Interobserver agreement for hemodynamic measurements was assessed using Bland-Altman analysis (Supplementary Figure S1). The analysis revealed a mean difference close to zero, with the majority of data points lying within the 95% limits of agreement. No systematic bias or proportional error was observed, indicating good reproducibility between the two observers. In addition, the intraclass correlation coefficient (ICC) for the primary outcome measure (WSSmax at the RICA-1 segment) was 0.939 (95% CI: 0.877–0.971, *p* < 0.001), indicating excellent interobserver reproducibility.

**Table 1:** Baseline characteristics of Low cardiovascular risk participants stratified by Serum TG level.

Characteristic	Total (*N* = 106)	Normal TG Group (TG <1.7 mmol/L, *N* = 59)	Elevated TG Group (TG 1.7–5.64 mmol/L, *N* = 20)	Severely Elevated TG Group (TG ≥5.65 mmol/L, *N* = 27)	Overall *p*-values	Normal vs. Elevated *p*-value	Normal vs. Severely Elevated *p*-value	Elevated vs. Severely Elevated *p*-value
Gender (male)	62 (58.4%)	22 (37.9%)	16 (80%)	23 (85.2%)	*P* < 0.001^*^	*P* < 0.001^*^	*P* < 0.001^*^	*P* = 0.986
Age (y)^a^	37.05 ± 8.17	34.34 ± 7.93	41.31 ± 7.22	39.51 ± 7.62	*P* < 0.001^*^	*P* < 0.001^*^	*P* = 0.07	*P* = 0.378
Presence of Smoking	44 (41.5%)	20 (33.8%)	14 (63.6%)	10 (37%)	*P* < 0.001^*^	*P* = 0.05^*^	*P* = 0.777	*P* = 0.025^*^
BMI (kg/m^2^)	23.28 ± 3.73	22.09 ± 3.46	25.89 ± 2.98	25.77 ± 2.36	*P* < 0.001^*^	*P* < 0.001^*^	*P* < 0.001^*^	*P* = 0.939
Heart Rate (bpm)	73.79 ± 11.17	72.06 ± 10.06	74.27 ± 11.82	77.25 ± 11.66	*P* = 0.124	*P* = 0.864	*P* = 0.569	*P* = 0.550
TC (mmol/L)	4.74 ± 0.96	4.25 ± 0.72	5.08 ± 0.90	5.60 ± 0.71	*P* < 0.001^*^	*P* < 0.001^*^	*P* < 0.001^*^	*P* = 0.021^*^
HDL-C (mmol/L)	1.15 ± 0.32	1.35 ± 0.22	1.14 ± 0.32	0.88 ± 0.15	*P* = 0.553	*P* = 0.470	*P* = 0.376	*P* = .0949
Drinking history (%)	42 (39.6%)	28 (48.27%)	7 (31.8%)	7 (25.9%)	*P* = 0.105	*P* = 0.332	*P* = 0.059	*P* = 0.501
Family history of CVD (%)	38 (35.9%)	24 (40.7%)	5 (25%)	9 (33.3%)	*P* = 0.428	*P* = 0.209	*P* = 0.516	*P* = 0.537
Waist weight (cm)	80.34 ± 14.48	78.50 ± 13.99	81.13 ± 14.48	83.79 ± 15.38	*P* = 0.283	*P* = 0.482	*p* = 0.118	*P* = 0.535

a Mean ± SD for continuous variables and percentage for categorical variables.

* Denotes statistical significance (*p* < 0.05).

BMI, body mass index; TG, triglyceride; TC, total cholesterol; HDL-C, high density lipoprotein cholesterol; CVD, cardiovascular disease.

**Figure 4:**
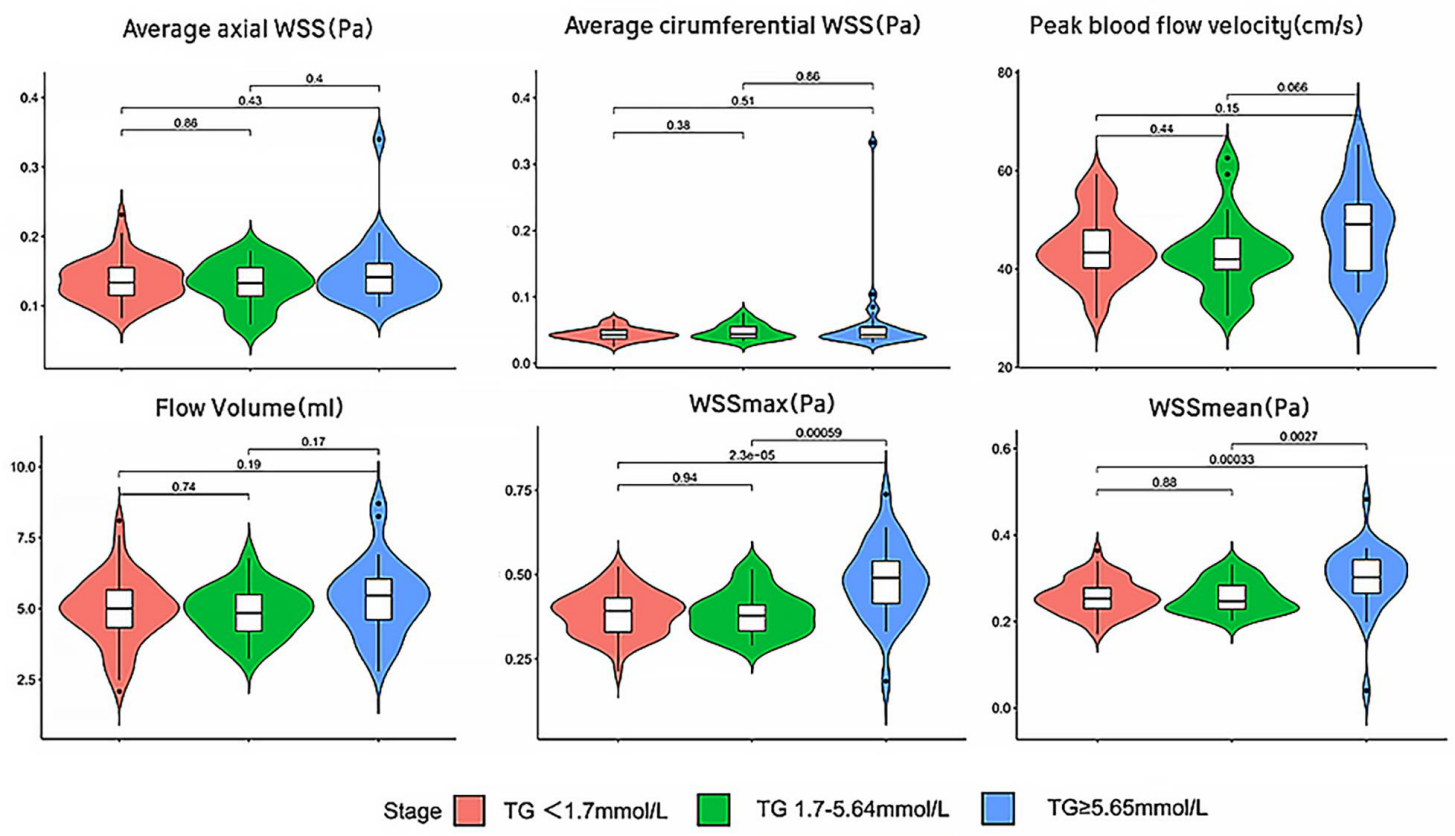
Statistical analysis of intergroup comparisons of carotid artery hemodynamic parameters obtained via 4D flow MRI. The figure shows significant statistical differences in WSSmax and WSSmean values across the normal TG level group, elevated TG group, and severely elevated TG group. Notably, the differences between the severely elevated TG group and the other two groups are prominent, while no significant difference is observed between the elevated TG group and the normal TG group.

Additionally, the hemodynamic parameters of the eight vascular segments were averaged to obtain a single representative value for each participant, which was then used for subsequent intergroup comparison. The results showed statistically significant differences in mean WSSmax between the severely elevated TG group and both the elevated TG group (*p* = 0.00059) and the normal TG group (*p* = 0.00000), with *P*-values < 0.001. No significant difference was observed between the elevated TG group and the normal TG group (*P* = 0.94). Similarly, the average WSSmean showed significant differences between the severely elevated TG group and both the elevated TG group (*p* = 0.0027) and the normal TG group (*P* = 0.00033), with *P*-values < 0.05. However, no significant difference was found between the elevated TG group and the normal TG group (*p* = 0.88). No statistically significant differences were observed in other hemodynamic parameters (see Figure 4).

### Multiple linear regression model and mixed-effects model

Two complementary multivariable approaches were used to evaluate the independent association between TG and hemodynamic parameters while adjusting for age, sex, total cholesterol, HDL-C, BMI, and smoking status.

In the multiple linear regression models based on per-participant averaged values, TG was independently associated with WSSmax (B = 0.011, *p* = 0.011) but not with WSSmean (*p* = 0.090). Age was negatively associated with peak velocity and WSSmax; male sex was associated with higher flow volume; BMI and smoking were significant predictors of WSSmean. Model R^2^ values ranged from 0.053 to 0.330 (full results in Supplementary Table S2).

In the linear mixed-effects models using segment-level data (eight segments per participant) with random intercepts for participant ID, TG remained significantly associated with WSSmax (B = 0.010, *p* < 0.001), WSSmean (B = 0.004, *p* = 0.004), flow volume (B = 0.090, *p* = 0.006), and average axial WSS (B = 0.003, *p* = 0.015), but not with peak velocity (*p* = 0.182) or circumferential WSS (*p* = 0.440). Smoking and BMI showed significant associations across multiple parameters, while TC and HDL-C were associated with selected outcomes.

**Figure 5:**
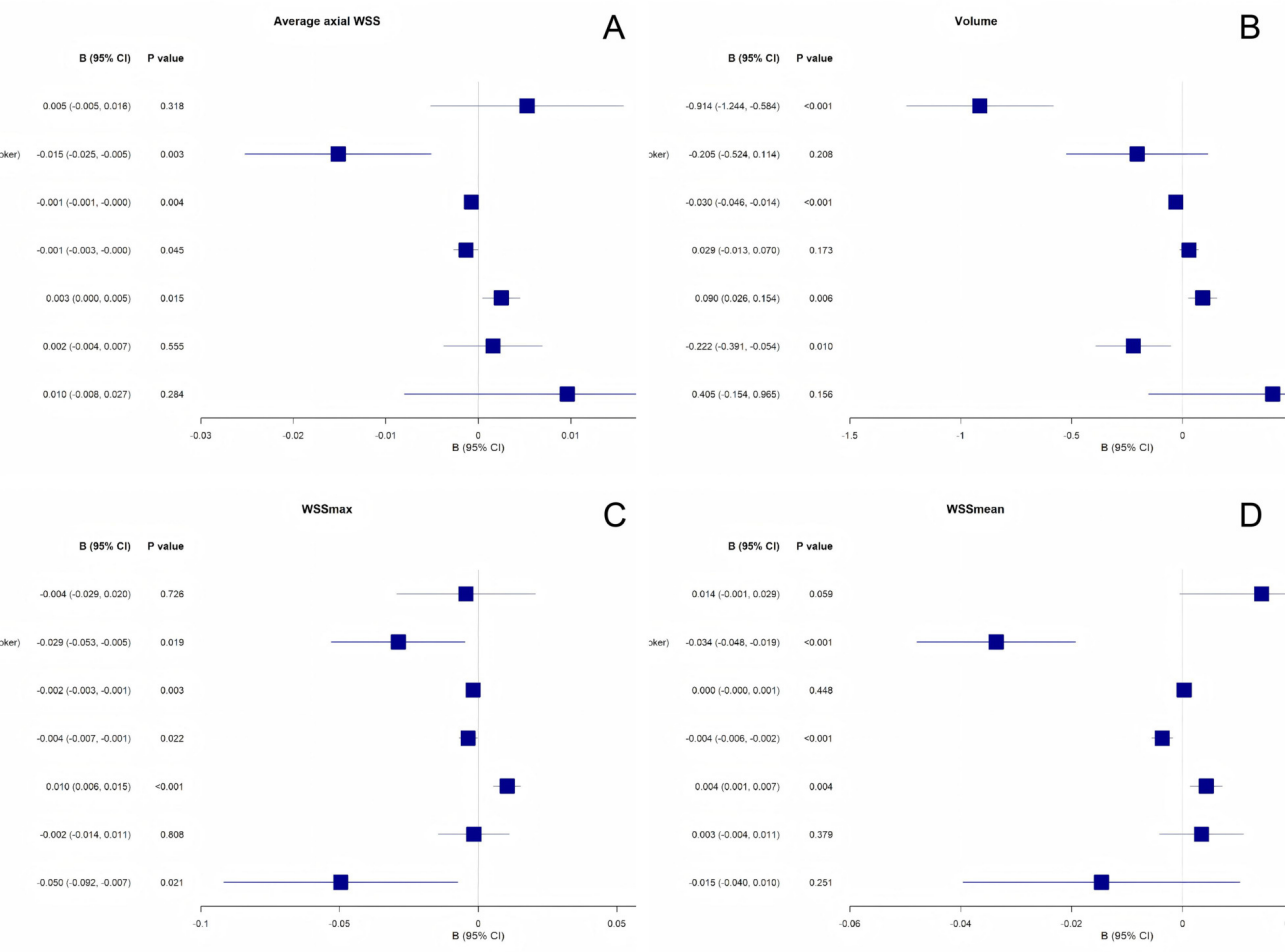
Mixed-effects model forest plots for carotid hemodynamic parameters. **(A)** WSSmax, **(B)** WSSmean, **(C)** Average axial WSS, **(D)** Flow volume. Adjusted for age, sex, BMI, smoking, TC, and HDL-C. Error bars represent 95% confidence intervals. TG was significantly associated with all four parameters (*p* < 0.05; see Supplementary Table S3 for full results).

Figure 5 presents forest plots of the mixed-effects model results for the four hemodynamic parameters significantly associated with TG. Detailed results of both regression models are provided in Supplementary Table S2 (linear regression) and Supplementary Table S3 (mixed-effects model).

## Discussion

Our findings demonstrate that, in a population with lower cardiovascular risk, significant differences in WSS-related parameters, particularly WSSmax and WSSmean, were observed across the three experimental groups, especially at the bifurcation of the bilateral internal carotid arteries (ICA-1). These differences were most prominent in participants with severely elevated TG levels (≥5.65 mmol/L/500 mg/dL), compared to those with elevated TG or normal TG levels (*p* < 0.001 for both comparisons). Elevated WSS represents a crucial hemodynamic parameter that characterizes the complex interactions between blood flow dynamics and vascular endothelial function (20). The observation that higher WSS values were found in the severely elevated TG group may seem to differ from the classical paradigm that low and oscillatory WSS promotes atherogenesis. One possible explanation is that the substantially higher peak blood flow velocity in the severely elevated TG group (43.95 cm/s in RICA-1) could contribute to the elevated WSS, suggesting a high-flow state rather than necessarily indicating primary vascular pathology. Previous research has also suggested that hypertriglyceridemia may be associated with physiological alterations such as increased blood viscosity and disturbed flow patterns (21, 22), which might also play a role. However, as we did not directly measure blood viscosity in this study (see Limitations), this proposed mechanism remains speculative and warrants further investigation. The observation of higher WSS in the severely hypertriglyceridemic group should be interpreted as a haemodynamic consequence of increased flow rather than a primary indicator of vascular injury. The higher flow volume and peak velocity noted in this group are consistent with a systemic high-flow state that may accompany hypertriglyceridemia. Although the exact underlying mechanism remains unclear, severe hypertriglyceridemia may reduce peripheral vascular resistance and facilitate systemic hyperperfusion, which could collectively explain the increased flow volume and peak velocity observed in the present low-risk population. Given the cross-sectional nature of this study, all reported associations should be interpreted as correlational and hypothesis-generating, not as evidence of causality.

The ICA-1 region exhibited the most pronounced hemodynamic alterations in response to elevated TG levels among all segments analyzed. While our planar analysis cannot directly elucidate the underlying mechanisms related to three-dimensional bifurcation geometry, the observed susceptibility of this proximal segment suggests that hemodynamic changes at the carotid bifurcation may be particularly sensitive to TG-associated modifications. It is important to note, however, that elevated WSS itself is not necessarily harmful; classical studies have established that low and oscillatory WSS promotes atherogenesis, whereas high laminar WSS is generally atheroprotective (23). That said, we hypothesize that under extreme conditions, sustained high WSS could contribute to endothelial activation through mechanotransduction pathways (24), although this has not been directly tested in our study. For example, recent work has shown that mechanical stimuli can activate pro-inflammatory pathways such as the TNK1/Tyk2/STAT1 axis in atherosclerosis (25). This offers a plausible, albeit still hypothetical, link between sustained high WSS and endothelial activation, which may deserve investigation in future studies. If this were the case, such mechanical stress could theoretically initiate a cascade of endothelial dysfunction, which could be an early event in the vascular pathophysiology associated with hypertriglyceridemia. Furthermore, while emerging evidence has suggested that hypertriglyceridemia might be associated with oxidative stress and inflammatory responses (26), we did not measure these pathways in our study. These potential mechanisms should therefore be considered as hypotheses to be tested in future investigations rather than as conclusions derived from our data.

In addition, the regression analyses in our study revealed a positive correlation between TG levels and both WSSmax and WSSmean. These findings are consistent with the possibility that elevated TG levels may be an important contributor to the observed changes in multiple hemodynamic parameters, particularly WSSmax and WSSmean, even in individuals without overt cardiovascular disease. This observation is consistent with prior research demonstrating that abnormal shear stress may influence gene expression in endothelial cells, which could in turn be associated with the release of pro-inflammatory cytokines and chemokines. If released, these inflammatory mediators might attract monocytes and lymphocytes to the vessel wall, potentially contributing to a localized inflammatory response. Moreover, it has been proposed that the altered flow patterns associated with elevated WSS could, under certain conditions, affect the endothelial layer, which might compromise vascular integrity and influence lipid deposition (27). Thus, elevated TG levels could be considered an early marker of endothelial stress, possibly mediated through WSS-related mechanisms, although this remains speculative. Age-related changes in carotid haemodynamics were also observed, consistent with prior studies using 4D Flow MRI (16). Even in individuals younger than 55 years, advancing age was associated with a decline in peak blood flow velocity and altered WSS parameters, suggesting that age remains an independent determinant of vascular function. It is important to note, however, that the effect sizes observed in our regression models were modest (e.g., TG coefficients of 0.010–0.090 for significant associations), and the R^2^ values were low (0.053–0.330). This indicates that TG alone explains only a small fraction of the variance in WSS. Therefore, while statistically significant, the clinical relevance of these small effect sizes in a low-risk population remains to be determined.

The modest regression coefficients between TG and WSS may stem from indirect mechanisms (e.g., TG influencing endothelial function rather than directly altering flow dynamics) or measurement limitations in capturing localized shear stress. To reconcile our finding of higher WSS in the severely hypertriglyceridemic group with the classical low-WSS atherogenic paradigm, two non-mutually exclusive possibilities exist: spatial heterogeneity (systemic high WSS may mask undetected low-WSS zones at bifurcations) or context-dependent pro-inflammatory effects of sustained high WSS, as suggested by mechanotransduction models (23, 24). Both possibilities, however, remain speculative and require direct experimental validation. Thus, while WSS may exert context-dependent roles, spatially resolved assessments are needed to clarify the relationship between local hemodynamics and vascular risk. Importantly, our cross-sectional design precludes any causal inference, and the observed associations should be interpreted as hypothesis-generating.

### Limitations

This study has several limitations. Firstly, the single-center, cross-sectional design inherently restricts the generalizability of our findings to broader population groups and limits our ability to establish temporal relationships. These methodological constraints highlight the need for future multicenter, longitudinal cohort studies to validate our observations and elucidate the temporal evolution of TG-WSS interactions. Second, although we hypothesized that elevated blood viscosity may mediate the relationship between hypertriglyceridemia and increased WSS based on previous literature (21, 22), we did not directly measure blood viscosity in this study. Therefore, the proposed mechanistic pathway remains speculative and requires confirmation in future studies incorporating direct viscosity measurements. Third, our study population primarily comprised individuals with low cardiovascular risk profiles, which necessitates cautious interpretation when considering potential extrapolation to high-risk populations. Fourth, 4D Flow MRI may underestimate true WSS due to limited spatial resolution and VENC settings, particularly in complex flow regions (20, 22). Fifth, we did not measure markers of insulin resistance or visceral adiposity; therefore, we cannot exclude that the observed TG-WSS association may partly reflect a broader metabolic phenotype. Additionally, the exclusion of 74 participants due to incomplete lipid panels or imaging data loss precluded a formal comparison between included and excluded participants, and potential selection bias cannot be excluded. *post-hoc* power analysis showed that while the overall three-group comparison was well powered (power > 0.999), the normal-vs.-elevated TG pairwise comparison was underpowered (power  = 0.05) due to the nearly identical WSSmax values, and the small sample size of the elevated TG group (*n* = 20) limits detection of intermediate effects.

## Conclusion

This study demonstrates significant differences in WSS-related hemodynamic parameters across TG levels, with the most pronounced changes in the proximal internal carotid artery region among individuals with severely elevated TG levels. These findings suggest that TG levels may influence carotid hemodynamics even in low cardiovascular risk populations.

## Data Availability

The datasets generated during this study are available from the corresponding author upon reasonable request.
