# Supplementary material for: Investigating association of triglycerides with hemodynamic parameters in patients with Low cardiovascular risk using 4D flow MRI
Source: Front Cardiovasc Med. 2026 Jun 24;13:1826181. doi: 10.3389/fcvm.2026.1826181 (PMC13341862; doi:10.3389/fcvm.2026.1826181)
Supplement: Supplementary file 1 [file Datasheet1.pdf]

Table S1. Hemodynamic Parameters of Carotid Artery Segments by TG Group

Artery	Parameter	TG <1.7 mmol/L (N=59)	TG 1.7–5.64 mmol/L (N=20)	TG ≥5.65 mmol/L (N=27)	p value
RCCA-M	Flow Volume (ml)	5.90 ± 1.55	6.31 ± 1.41	6.06 ± 1.72	0.669
	Peak blood flow velocity (cm/s)	53.10 ± 10.72	51.16 ± 11.85	55.04 ± 13.93	0.247
	Average axial WSS (Pa)	0.14 ± 0.05	0.14 ± 0.04	0.17 ± 0.06	0.048
	Average circumferential WSS (Pa)	0.04 ± 0.01	0.04 ± 0.01	0.06 ± 0.07	0.762
	WSSmax (Pa)	0.50 ± 0.14	0.50 ± 0.12	0.57 ± 0.15	0.033
	WSSmean (Pa)	0.30 ± 0.08	0.30 ± 0.05	0.35 ± 0.12	0.038
RCCA	Flow Volume (ml)	6.47 ± 1.72	6.84 ± 1.30	6.45 ± 1.76	0.784
	Peak blood flow velocity (cm/s)	49.45 ± 10.18	48.26 ± 11.82	52.18 ± 9.58	0.320
	Average axial WSS (Pa)	0.13 ± 0.04	0.12 ± 0.04	0.16 ± 0.06	**0.009**

	Average circumferential WSS (Pa)	0.04 ± 0.01	0.04 ± 0.01	0.07 ± 0.13	0.097
	WSSmax (Pa)	0.42 ± 0.13	0.42 ± 0.09	0.52 ± 0.13	**0.001**
	WSSmean (Pa)	0.28 ± 0.07	0.27 ± 0.05	0.32 ± 0.08	**0.007**
RICA-1	Flow Volume (ml)	4.30 ± 1.60	3.61 ± 1.44	5.21 ± 2.52	0.041
	Peak blood flow velocity (cm/s)	36.95 ± 7.17	32.32 ± 8.09	43.95 ± 10.80	**<0.001**
	Average axial WSS (Pa)	0.12 ± 0.04	0.12 ± 0.04	0.14 ± 0.06	0.592
	Average circumferential WSS (Pa)	0.04 ± 0.01	0.04 ± 0.01	0.05 ± 0.06	0.116
	WSSmax (Pa)	0.29 ± 0.10	0.29 ± 0.10	0.44 ± 0.14	**<.001**
	WSSmean (Pa)	0.20 ± 0.06	0.20 ± 0.06	0.28 ± 0.08	**<.001**
RICA-2	Flow Volume (ml)	3.51 ± 1.19	3.48 ± 1.41	3.99 ± 1.69	0.760
	Peak blood flow velocity (cm/s)	40.48 ± 8.26	42.18 ± 15.99	39.07 ± 16.31	0.249

	Average axial WSS (Pa)	0.14 ± 0.06	0.13 ± 0.05	0.13 ± 0.06	0.743
	Average circumferential WSS (Pa)	0.05 ± 0.03	0.07 ± 0.04	0.06 ± 0.07	0.371
	WSSmax (Pa)	0.32 ± 0.09	0.35 ± 0.10	0.43 ± 0.14	**0.002**
	WSSmean (Pa)	0.25 ± 0.07	0.26 ± 0.07	0.28 ± 0.08	0.112
LCCA-M	Flow Volume (ml)	5.14 ± 1.43	5.91 ± 1.19	6.05 ± 1.41	**0.004**
	Peak blood flow velocity (cm/s)	53.62 ± 10.73	52.51 ± 13.75	57.55 ± 13.68	0.258
	Average axial WSS (Pa)	0.16 ± 0.06	0.15 ± 0.06	0.15 ± 0.08	0.516
	Average circumferential WSS (Pa)	0.04 ± 0.02	0.04 ± 0.01	0.05 ± 0.07	0.301
	WSSmax (Pa)	0.50 ± 0.11	0.52 ± 0.17	0.54 ± 0.12	0.388
	WSSmean (Pa)	0.31 ± 0.07	0.31 ± 0.09	0.32 ± 0.09	0.422
LCCA	Flow Volume (ml)	5.65 ± 1.49	6.58 ± 1.42	6.54 ± 1.64	**0.013**

	Peak blood flow velocity (cm/s)	48.47 ± 9.54	47.78 ± 11.50	53.56 ± 11.09	0.087
	Average axial WSS (Pa)	0.13 ± 0.04	0.12 ± 0.04	0.15 ± 0.07	0.207
	Average circumferential WSS (Pa)	0.04 ± 0.01	0.03 ± 0.01	0.06 ± 0.08	0.301
	WSSmax (Pa)	0.44 ± 0.10	0.42 ± 0.13	0.51 ± 0.14	0.025
	WSSmean (Pa)	0.28 ± 0.05	0.29 ± 0.06	0.31 ± 0.09	0.088
LICA-1	Flow Volume (ml)	4.64 ± 1.60	3.65 ± 1.42	4.79 ± 1.93	0.052
	Peak blood flow velocity (cm/s)	33.81 ± 6.26	31.25 ± 7.06	43.20 ± 10.74	**<.001**
	Average axial WSS (Pa)	0.12 ± 0.05	0.13 ± 0.06	0.14 ± 0.08	0.939
	Average circumferential WSS (Pa)	0.04 ± 0.02	0.05 ± 0.02	0.06 ± 0.06	0.092
	WSSmax (Pa)	0.29 ± 0.08	0.24 ± 0.09	0.42 ± 0.14	**<.001**
	WSSmean (Pa)	0.21 ± 0.06	0.17 ± 0.07	0.28 ± 0.08	**<.001**

LICA-2	Flow Volume (ml)	3.71 ± 1.13	3.28 ± 0.92	3.83 ± 1.39	0.385
	Peak blood flow velocity (cm/s)	39.61 ± 7.59	37.44 ± 8.89	37.48 ± 8.92	0.568
	Average axial WSS (Pa)	0.15 ± 0.08	0.14 ± 0.07	0.14 ± 0.07	0.820
	Average circumferential WSS (Pa)	0.06 ± 0.03	0.07 ± 0.04	0.06 ± 0.05	0.405
	WSSmax (Pa)	0.34 ± 0.09	0.30 ± 0.10	0.40 ± 0.11	**0.008**
	WSSmean (Pa)	0.25 ± 0.07	0.23 ± 0.07	0.27 ± 0.08	0.298

TG,triglyceride; Wall Shear Stress （WSS）

Data are presented as mean ± SD. P-values are from Kruskal-Wallis test comparing the three TG groups. Benjamini-Hochberg false discovery rate (FDR) correction was applied across all 48 comparisons (8 segments × 6 parameters). Bold indicates p-values that remained significant after FDR correction (FDR < 0.05).

Table S2. Detailed Results of the Multiple Linear Regression Model

**Parameter**	**Variable**	**Coefficient(B)**	**p value**	**Intercept**	**R^2**
Flow Volume	Gender	0.831	0.007*	5.497	0.200
	Age	-0.032	0.029*		

Parameter	Variable	Coefficient(B)	p value	Intercept	R^2
	TG	0.083	0.157		
	TC	-0.202	0.189		
	HDL	0.340	0.504		
	BMI	0.020	0.597		
	Smoking	0.221	0.447		
Peak blood flow velocity	Gender	2.103	0.245	68.655	0.330
	Age	-0.411	<.001*		
	TG	0.284	0.417		
	TC	0.145	0.875		
	HDL	-4.879	0.112		
	BMI	-0.278	0.221		
	Smoking	3.980	0.024*		
Average axial WSS	Gender	-0.003	0.731	0.178	0.115
	Age	-0.001	0.070		
	TG	0.002	0.227		

Parameter	Variable	Coefficient(B)	p value	Intercept	R^2
	TC	0.000	0.983		
	HDL	0.012	0.471		
	BMI	-0.001	0.271		
	Smoking	0.018	0.062		
Average circumferential WSS	Gender	-0.010	0.251	0.061	0.053
	Age	0.000	0.606		
	TG	0.001	0.597		
	TC	0.003	0.479		
	HDL	-0.014	0.327		
	BMI	0.000	0.974		
	Smoking	0.003	0.728		
WSSmax	Gender	0.008	0.698	0.578	0.262
	Age	-0.002	0.050		
	TG	0.011	0.011*		
	TC	-0.004	0.726		

Parameter	Variable	Coefficient(B)	p value	Intercept	R^2
	HDL	-0.041	0.266		
	BMI	-0.003	0.270		
	Smoking	0.028	0.190		
WSSmean	Gender	-0.015	0.297	0.326	0.185
	Age	0.000	0.629		
	TG	0.005	0.090		
	TC	0.004	0.623		
	HDL	-0.013	0.582		
	BMI	-0.004	0.040*		
	Smoking	0.033	0.015*		

B, unstandardized regression coefficient. All models adjusted for age, sex, TG, TC, HDL-C, BMI, and smoking status. Significant associations ($p < 0.05$) are marked with an asterisk (*).

Table S3. Detailed Results of the Mixed Effects Model

Parameter	Variable	Coefficient(B)	p value
Flow Volume	Gender (Female)	-0.914	<0.001*
	Smoking (No)	-0.205	0.208
	Age	-0.030	<0.001*
	BMI	0.029	0.173
	TG	0.090	0.006*
	TC	-0.222	0.010*
	HDL	0.405	0.156
Peak blood flow velocity	Gender (Female)	-1.791	0.105
	Smoking (No)	-3.952	<0.001*
	Age	-0.394	<.001*
	BMI	-0.287	0.039*
	TG	0.285	0.182
	TC	0.189	0.737
	HDL	-4.881	0.009*
Average axial WSS	Gender (Female)	0.005	0.318

Parameter	Variable	Coefficient(B)	p value
	Smoking (No)	-0.015	0.003*
	Age	-0.001	0.004*
	BMI	-0.001	0.045*
	TG	0.003	0.015*
	TC	0.002	0.555
	HDL	0.010	0.284
Average circumferential WSS	Gender (Female)	0.009	0.012*
	Smoking (No)	-0.002	0.586
	Age	0.000	0.362
	BMI	0.000	0.951
	TG	0.001	0.440
	TC	0.003	0.148
	HDL	-0.014	0.026*
WSSmax	Gender (Female)	-0.004	0.726
	Smoking (No)	-0.029	0.019*
	Age	-0.002	0.003*

Parameter	Variable	Coefficient(B)	p value
	BMI	-0.004	0.022*
	TG	0.010	<0.001*
	TC	-0.002	0.808
	HDL	-0.050	0.021*
WSSmean	Gender (Female)	0.014	0.059
	Smoking (No)	-0.034	<0.001*
	Age	0.000	0.448
	BMI	-0.004	<0.001*
	TG	0.004	0.004*
	TC	0.003	0.379
	HDL	-0.015	0.251

B, unstandardized fixed-effects coefficient. Mixed-effects models included random intercepts for participant ID and vascular segment as a repeated measure with a diagonal covariance structure. All models adjusted for age, sex, TG, TC, HDL-C, BMI, and smoking status. Significant associations ($p < 0.05$) are marked with an asterisk (*).
